# A Potentially Dangerous Industrial Projectile Lodged in the Leg of a Steel Factory Worker

**DOI:** 10.7759/cureus.17870

**Published:** 2021-09-10

**Authors:** Ankit Khurana, Vishal Jain, Shailendra C Gupta, Kuldeep Malik, Sudhir Gupta

**Affiliations:** 1 Orthopaedics, ESI Hospital Rohini, Delhi, IND; 2 Anaesthesiology, ESI Hospital Rohini, Delhi, IND

**Keywords:** dystrophic calcification, injury biomechanics, foreign body, factory injury, employment injury

## Abstract

Penetrating injuries due to fragments energized by an explosive event are life/limb-threatening and are associated with poor clinical and functional outcomes. Penetrating injuries are commonly inflicted in attacks with explosive devices. The extremities, especially the leg, are the most commonly affected body areas, presenting a high risk of infection, slow recovery, and the threat of amputation. This report presents a case of a young factory worker who sustained an injury to the leg with a foreign body lodged near the neuro-vascular bundle.

A 44-year-old gentleman sustained a projectile injury while working in a stainless steel factory from the rula (steel rolling) machine with a foreign body getting lodged in the leg in March 2019. He was initially managed with wound care and didn't report any functional impairment. Gradually patient developed numbness and claudication symptoms of the foot over the next couple of years. He was subsequently operated on in 2021 for removal of the stainless steel foreign body encased in dystrophic calcification close to the tibial nerve and posterior tibial vessels. Interestingly the entry point of the foreign body was on the anterolateral aspect of the leg. The foreign body was removed using the postero-lateral approach to the tibia with careful dissection close to the neurovascular bundle. At a follow-up of 3 months, the patient is symptom-free with significant improvement of limb function.

The authors propose that the foreign body crossed the interosseous membrane to get lodged close to the posterior tibial neurovascular bundle. In such a scenario, the patient was extremely lucky to have survived an amputation or significant functional injury of the limb. Proper protective equipment is needed not only for the torso but also for extremities to protect industrial workers from such limb-threatening injuries. Moreover, primary care physicians should be sensitised for the proper management of such injuries.

## Introduction

Energized fragment penetration is a common injury modality among blast injuries in factories. Penetrating injuries due to fragments energized by an explosive event can be life as well as limb-threatening [1]. These are often associated with poor clinical and functional outcomes due to the significant kinetic energy of these projectiles [2]. There is a wide range of factors that can potentially affect injury outcomes, such as impact velocity, projectile mass, and shape [3-5]. The majority of such ballistics research currently focuses on gunshot wounds, and limited objective experimental evidence exists to quantify the potential injurious mechanisms of energized fragments from factory equipment.

This report brings to light one such interesting and potentially limb-threatening injury to the leg of a young factory worker employed in a semi-urban steel factory in India who sustained a projectile injury to the leg with a foreign body lodged near the neurovascular bundle that over time caused claudication symptoms.

## Case presentation

**Figure 1:**
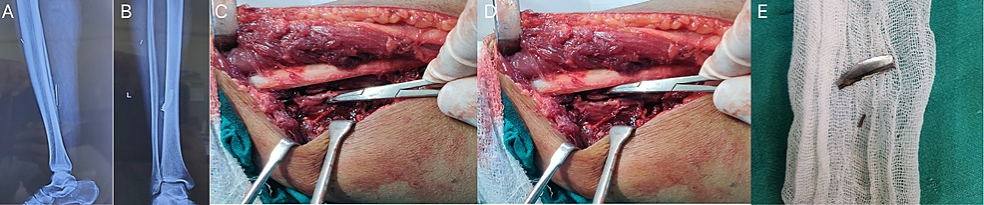
Radiographs, preoperative images, and extracted foreign body A 44-year-old male with a metal foreign body present in the posterolateral aspect of the tibia depicted radiographically (1A and B). Preoperative images (1C and D) using the posterolateral approach show close proximity of the foreign body (marked by haemostat in 1D) to the posterior tibial vessels as pointed by haemostat in figure 1C. Following extraction, the foreign body is seen to be a sharp steel projectile of size 3x1 cm (1E)

A 44-year-old gentleman sustained a projectile injury to the leg in March 2019 while working in a stainless steel factory from a rula (steel rolling) machine. He was initially managed with wound care by a local practitioner and didn't report any functional impairment. No radiological investigation was performed at this time. Gradually over the next two years, the patient began developing numbness and claudication symptoms of the foot which worsened over time. The patient presented to the authors in January 2021 when the first radiographs were performed, which revealed a foreign body lodged in the leg (Figure 1). 

He was subsequently operated and a posterolateral approach was chosen to extract the foreign body. A 10 cm longitudinal incision was taken over the lateral border of the gastrocnemius muscle centred over the radiographic location of the foreign body. The internervous plane which lies between the gastrocnemius, soleus, and flexor hallucis longus muscles (tibial nerve) and the peroneal muscles (superficial peroneal nerve) was used taking care not to injure the short saphenous vein and peroneal artery.

The stainless steel foreign body was found encased in dystrophic calcification in close proximity to the tibial nerve and posterior tibial vessels (Figure 1). The foreign body was subsequently removed using careful dissection close to the neuro-vascular bundle and was found to be sharp stainless steel shrapnel of size 3x1cm (Figure 1). Interestingly, the entry point of the foreign body was on the anterolateral aspect of the leg. At a follow-up of 3 months, the patient was symptom-free with significant improvement of limb function.

## Discussion

Based on our literature search, this study is the first to evaluate injury due to penetration of steel factory projectile to the musculoskeletal system. Foreign bodies such as bullets, shotgun pellets, and shrapnel can cause clinical symptoms by mechanical compression [6], lumen obstruction [7], irritation of nearby structures [8], systemic heavy metal intoxication [9], or tumour formation [10,11]. Chong et al. have proposed the use of particle-based computational methods to simulate injury and haemorrhage in the human body, and their study demonstrates a significant haemorrhage following a ballistic injury to the leg [12].

Similar to our findings, an ischemic syndrome has been demonstrated in a 5-year-old girl with a gun-shot wound to the shoulder with an arterial embolus at the elbow leading to ischaemic symptoms [13]. There have also been reports of cardiac embolisation of metallic foreign bodies which are perivascular and have been retrieved by the trans-femoral route [14]. A similar report presents a pulmonary embolization of one such metallic foreign body, further emphasizing the dangerous nature of peri-vascular foreign bodies [15].

Additionally, projectile foreign bodies are known to cause chronic heavy metal toxicity due to the release of metal into the bloodstream [16]. Usually, metallic objects embedded into the soft tissue become encapsulated and do not release metals into the systemic circulation. However, there are exceptions to this rule [8]. Missiles close to the bone, [17], joints, and intervertebral disks are continuously bathed with synovial fluid, which eventually washes off lead from the bullets resulting in systemic toxicity [18,19]. Females are more vulnerable to this form of toxicity which can then lead to neuropathy [16,20].

**Figure 2:**
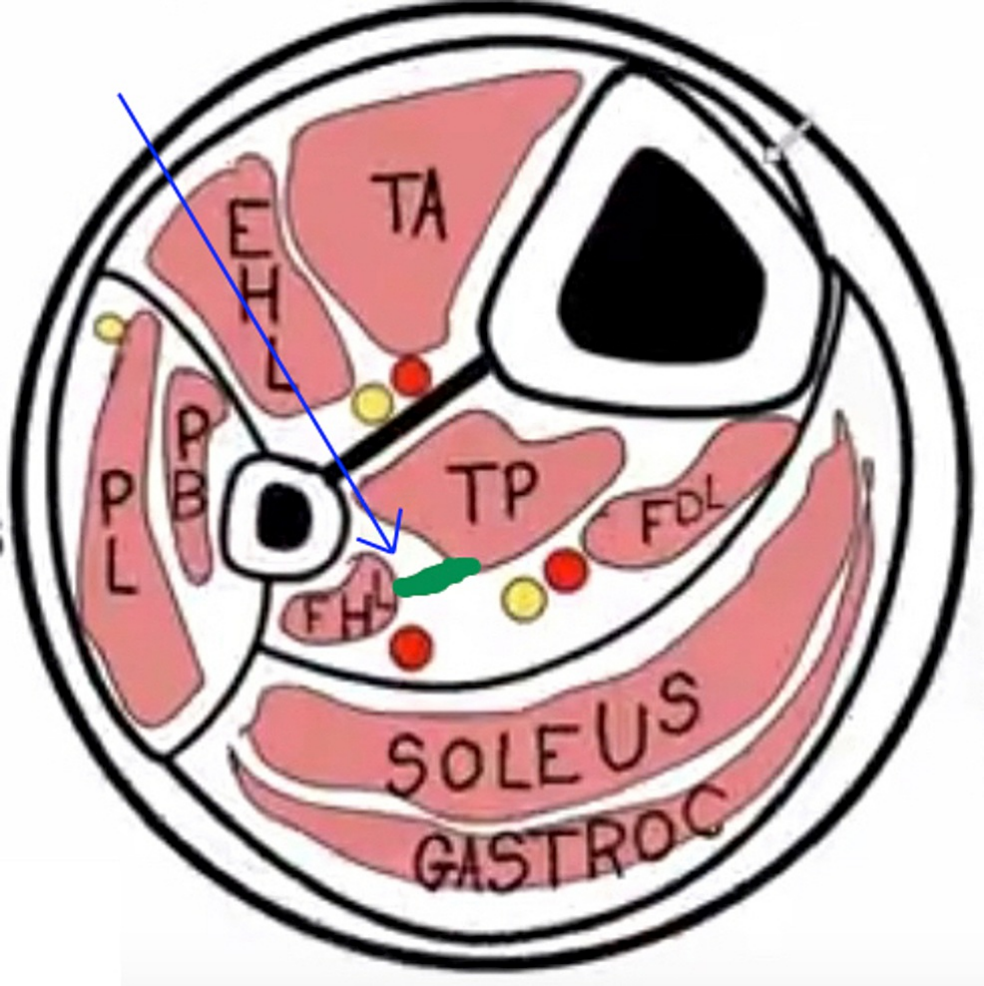
Diagram of the proposed trajectory of the projectile Showing the proposed trajectory of the projectile (blue arrow) and the foreign body (depicted in green) getting lodged in close proximity to the posterior tibial vessels and tibial nerve.

The authors propose that the projectile narrowly missed the anterior tibial neurovascular structures, including the deep peroneal nerve and anterior tibial artery and vein and crossed the interosseous membrane to lodge in close proximity to the posterior tibial artery and tibial nerve (Figure 2). The foreign body pressed on these structures to cause claudication like features. As the patient didn't have an infection of the wound or a significant loss of significant function, he ignored the claudication symptoms, which gradually increased over the next two years. With time, as the symptoms grew, the patient reported to us. Following removal, the patient noticed an immediate relief of symptoms, further proving the cause of claudication as the foreign body. 

## Conclusions

Keeping the above-mentioned risks in mind, proper protective equipment is needed not only for the torso but also for extremities to protect industrial workers from such limb-threatening injuries. Primary care physicians should be sensitised to these industrial injuries so that they can be properly evaluated and managed in primary care settings. Our findings can be used to predict the dangers due to industrial projectile trauma, instigating the development of situation-appropriate personal protective equipment as well as initiate implementation of mitigating strategies to avoid such mishaps in the future.
